# Mechanistic Insight into the T_H_1-Biased Immune Response to Recombinant Subunit Vaccines Delivered by Probiotic Bacteria-Derived Outer Membrane Vesicles

**DOI:** 10.1371/journal.pone.0112802

**Published:** 2014-11-26

**Authors:** Joseph A. Rosenthal, Chung-Jr. Huang, Anne M. Doody, Tiffany Leung, Kaho Mineta, Danielle D. Feng, Elizabeth C. Wayne, Nozomi Nishimura, Cynthia Leifer, Matthew P. DeLisa, Susana Mendez, David Putnam

**Affiliations:** 1 Department of Biomedical Engineering, Cornell University, Ithaca, NY, 14853, United States; 2 School of Chemical and Biomolecular Engineering, Cornell University, Ithaca, NY, 14853, United States; 3 Department of Animal Science, Cornell University, Ithaca, NY, 14853, United States; 4 College of Veterinary Medicine, Cornell University, Ithaca, NY, 14853, United States; Seoul National University College of Pharmacy, Republic of Korea

## Abstract

Recombinant subunit vaccine engineering increasingly focuses on the development of more effective delivery platforms. However, current recombinant vaccines fail to sufficiently stimulate protective adaptive immunity against a wide range of pathogens while remaining a cost effective solution to global health challenges. Taking an unorthodox approach to this fundamental immunological challenge, we isolated the TLR-targeting capability of the probiotic *E. coli* Nissle 1917 bacteria (EcN) by engineering bionanoparticlate antigen carriers derived from EcN outer membrane vesicles (OMVs). Exogenous model antigens expressed by these modified bacteria as protein fusions with the bacterial enterotoxin ClyA resulted in their display on the surface of the carrier OMVs. Vaccination with the engineered EcN OMVs in a BALB/c mouse model, and subsequent mechanism of action analysis, established the EcN OMV’s ability to induce self-adjuvanted robust and protective humoral and T_H_1-biased cellular immunity to model antigens. This finding appears to be strain-dependent, as OMV antigen carriers similarly engineered from a standard K12 *E. coli* strain derivative failed to generate a comparably robust antigen-specific T_H_1 bias. The results demonstrate that unlike traditional subunit vaccines, these biomolecularly engineered “pathogen-like particles” derived from traditionally overlooked, naturally potent immunomodulators have the potential to effectively couple recombinant antigens with meaningful immunity in a broadly applicable fashion.

## Introduction

The adaptive immune response to recombinant subunit antigens is enhanced by improved vaccine delivery system [1]–[4]. Increasingly, this success has come from innovations in the design of synthetic or biologically-derived nanoparticle antigen carriers [4]–[6]. Nanoparticle vaccine carriers can mimic the natural interaction between immune cells and recombinant antigens that extend beyond simple antigen identification [7], and allow more efficient and targeted dissemination of the antigen to key immune cell population [8]. With this success, however, it is evident that the induction of a directed T_H_1-biased cellular response against recombinant subunit vaccines remains challenging [9].

To address the limited T_H_1-biased response to subunit vaccines, and the unmet clinical needs they represent, we considered their delivery by vesicles derived from the outer membrane of probiotic Gram-negative bacteria, also known as OMVs. OMV-based vaccines produced directly from pathogens, in particular *Neisseria meningitides*, successfully prevent disease [10], and the literature indicates that OMVs have adjuvant capabilities that can help to direct immune responses [11]. There is compelling evidence that bacterial OMVs – although non-infectious – retain much of the capacity of live bacteria to actively engage both the innate and adaptive immune systems through a host of surface displayed and soluble-factor intracellular pathogen-associated molecular patterns (PAMPs) [12]–[14]. These molecular characteristics have inspired recent work with multi-strain meningococcal OMV vaccines [15] as well as cross-pathogen antigen presentation [16], and indicate that such natural adjuvancy can achieve immune responses unachievable with standard subunit vaccines.

Noting that the untapped potential of recombinant OMVs aligned well with key limitations of subunit vaccines, we investigated whether a T_H_1-biased recombinant vaccine delivery platform could be created from OMVs derived from Gram-negative probiotic bacteria. During colonization of the human digestive tract, certain probiotic bacteria suppress local immunity by modulating resident gut-associated lymphoid tissue cytokine activity [17]. Noting that one of the most consistent deficiencies in modern vaccines is the ability to stimulate cellular immunity, we sought probiotic bacteria whose immunomodulatory capacity was characterized by potent interactions with cell types crucial for T_H_1-biased immunity. Previous work in the field [18] has highlighted the potential for OMVs to induce such responses – our focus was therefore on optimizing, enhancing and understanding this inherent phenomenon through source strain selection to be robust enough to carry over to specifically directed immune responses to exogenous antigens. Specifically, we focused on the probiotic *E. coli* strain Nissle 1917 (EcN), which achieves this phenomenon in part through specifically targeting T-leukocytes for cell cycle and regulatory disruption via PAMP-dependent mechanisms that are specifically enriched in EcN [19], [20]. Such potent suppressive capacity does not make EcN, or any probiotic bacteria, an obvious candidate for vaccine applications. However, in marked contrast to the highly immunosuppressive bacteria themselves, OMVs derived from such Gram-negative probiotic bacteria would lack the predominantly secretory immunosuppressive capability of the intact organism while retaining the surface-displayed immunostimulatory PAMPs and, in EcN’s case, the rare capacity to actively bind to and stimulate T-cell TLRs [19]. Therefore, we hypothesized that OMVs comprised of the uniquely immunostimulatory EcN outer membrane could effectively leverage the probiotic strain’s distinctive ability to engage and activate key innate and adaptive immune cells [21], while simultaneously enhancing the natural adjuvanting mechanisms through which OMVs interact with immune cells and effectively mimic intracellular pathogens (Fig. S1 in File S1). If this hypothesis were validated, the resulting pathogen-like particles would have the potential to robustly couple carrier-presented recombinant exogenous antigens with both humoral and cellular immune responses.

While subunit antigen vaccines only rarely produce a potent, T_H_1-biased response, a pathogen-like particle used as a biomimetic antigen carrier can fundamentally address this deficiency in modern vaccine technology. Here, we show that the immunization of BALB/c mice with EcN OMVs engineered to surface display exogenous model subunit antigens induces robust antigen-specific humoral and cellular immunity. More importantly, both the development of humoral immunity through antibody class selection as well as the preferential development of specific CD4/CD8-positive T-cell cytokine expression phenotypes strongly indicates the induction of antigen-specific T_H_1-biased immunity. Mechanistic analysis of this phenomenon by the EcN OMVs reveals an immune reaction specific to the EcN strain that makes use of their unique combination of PAMP-TLR interactions, mediated through stimulation of both innate and adaptive immune cells. These results indicate an effectively engineered isolation of probiotic bacterial immunoactivity that has a previously unappreciated capacity as a scalable [11] and broadly applicable vaccine delivery carrier for challenging pathogen targets requiring strong, targeted T_H_1-mediated immunity.

## Materials and Methods

### Mice and cell cultures

BALB/c wild-type mice (8-week old females) were used as the subjects of all vaccine trials and as the source of all *in vitro* culture of primary cells. Experiments were approved by the Institutional Animal Care and Use Committee at Cornell University (Ithaca, NY), protocol number 2009-0096. For the macrophage knockout studies, MyD88, or TLR4, deficient macrophage cell lines (BEI resources) were cultured in Dulbeco’s modified essential medium supplemented with 10% (v/v) low endotoxin fetal bovine serum, 2 mM L-glutamine, 1 mM sodium pyruvate, 10 mM HEPES (complete DMEM) and 10 ug/ml ciprofloxacin [22]. Cells were stimulated with OMVs, or 100 ng/ml purified LPS as a control. For the TLR reconstitution assay, human embryonic kidney cells (HEK293) from the Leifer laboratory were cultured in complete DMEM supplemented with 50 U/ml penicillin and 50 ug/ml streptomycin. Cells were co-transfected in 96 well plates using TransIT (Muris) with the indicated TLRs, 5x-NF-kB-luciferase, and control vector to equal 200 ng/well total DNA. Cells were treated with OMVs, or PAM3CSK4 (TLR2), poly(I:C) (TLR3), LPS (TLR4), flagellin (TLR5), CpG DNA (TLR9), or profilin (TLR11). Cell lysates (5x Lysis buffer, Promega) were assayed for luciferase activity using the luciferase substrate as previously described [23].

### Bacterial Strain Mutations

*nlpI* and *lpxM* mutations were generated using P1 transduction of the *nlpI*::kan and *lpxM*::kan alleles, creating strains JH1 (Δ*nlpI*) and JH1-*LpxM* (Δ*nlpI*Δ*lpxM*), from the Keio collection [24]. Briefly, *E. coli* Nissle 1917 bacteria were grown overnight and incubated with P1 lysates from appropriate Keio single mutants, as described elsewhere [25]. Transduction was performed using 50 mM CaCl_2_ for 20 min at 37°C and 1 M Na-citrate for 40 min at room temp, and selective plating was done using medium containing 20 mM sodium citrate.

### Preparation of *E. coli* OMVs

OMVs were grown and purified in accordance with previously established procedure [26]. Briefly, plasmid pBAD-ClyA-GFP, containing an arabinose promoter, was transformed via heat shock into chemically competent preparations of *E. coli* vesicle-overproducing strains JC8031 (EcJ) [27], JH1, and JH1-*LpxM*, and selected in LB-chloramphenicol or LB-kanamycin medium, respectively. Flasks containing 250 mL of medium were inoculated with overnight culture and allowed to grow at 37°C and 230 rpm until the OD_600_ reached ∼0.5. Protein expression was induced at this point by addition of L-arabinose to a final concentration of 0.2%. Cell-free culture supernatants were collected 12 hours after induction (following centrifugation at 5000 g for 15 min) and then filtered through a 0.45 µm filter. Vesicles were isolated from all other soluble components by fractionation via ultracentrifugation (Beckman-Coulter; Ti SW28 rotor, 26000×g, 3 h, 4°C), resuspended in PBS, then stored at −20°C.

### Characterization of OMV Vaccines

Visual characterization was conducted using a LEO 1550 FESEM, a FEI Tecnai F20 TEM, and an Olympus BX41 for SEM, TEM, and fluorescent microscopy, respectively. Additional quantitative fluorescence analysis [28] was conducted on a Gemini EM microplate spectrofluorometer (Molecular Devices). Dynamic light scattering measurements were conducted using a Nanosizer Nano ZS (Malvern Instruments), using the refractive index and viscosity of water (1.333 and 0.90 cP). Further protein characterization was conducted using the bicinchoninic-acid assay (BCA Protein Assay; Pierce) and anti-GFP Western blot (antibodies from Invitrogen and Jackson Immunoresearch), with control proteins grown in DH5 bacterial culture as described previously [29]. LPS content was analyzed using a KDO colorimetric assay [30]–[31] and proteomics were conducted using the isobaric tag for relative and absolute quantitation, or iTRAQ, method. Glysosylation analysis was performed using chromatography-facilitated mass spectrometry.

### Mouse Immunizations

Ten groups (n = 10, each group) of 8-week old BALB/c mice (Charles River Laboratories) each were immunized s.c. with PBS (100 µL)containing purified protein or OMV preparations as described. The ten treatment groups were immunized with, respectively: PBS, 2.5 µg GFP, 2.5 µg ClyA, 2.5 µg GFP and ClyA mixture, 2.5 µg ClyA-GFP fusion, 2.5 µg alum and ClyA-GFP mixture (Alyhydrogel, 1.3% aluminum hydroxide [m/w]), and recombinant fluorescent equivalents of EcJ OMVs + ClyA-GFP, EcJ OMVs (with no recombinant antigen, or “empty”), EcN OMVs + ClyA-GFP, and EcN OMVs (empty). Two doses of vaccine (priming dose and boosting dose) were administered 4 weeks apart. Blood was collected from the mandibular sinus immediately before and 2 weeks after the first immunization, immediately before the boosting dose, and at 2 and 4 weeks after the boosting dose. Terminal splenectomies were performed on half (n = 5) of all ten groups immediately before administration of the boosting dose and on the other half (n = 5) following the final blood collection.

### Cell isolation and culture

For bone marrow-derived macrophages and dendritic cells (DCs), bone marrow was obtained from mouse femurs and grown for 6 to 8 days in RPMI 1640 in the presence of 10% L929 conditioned medium [32] and collected 7 days later by scraping. Bone marrow-derived DCs were cultured in the presence of GM-CSF (20 ng/ml) and collected 6–8 days after culture [33]. For the splenic T-cells and B-cells, spleens were harvested, mechanically homogenized, and filtered through a 100 µm cell strainer (Falcon). Erythrocytes were lysed with cold ACK lysing buffer (Cellgro) for 5 min, and the cell suspension was washed with complete medium. T cells were purified with negative selection enrichment columns (R&D Systems) following the manufacturer’s recommendations. B cells were enriched by negative selection using magnetic isolation kits, as per manufacturer’s instructions (Miltenyi Biotec). This process resulted in cell purities of >97% as determined by flow cytometry.

### ELISA for Serum Antibody Response

Polystyrene microtiter 96-well plates (Maxisorp; Nunc Nalgene) were coated with GFP (5 µg/ml in carbonate buffer, pH 9.6) and incubated overnight at 4°C. Plates were blocked with 3% BSA in PBS containing 0.05% Tween-20 (PBST). Samples were serially diluted 2-fold in blocking buffer in a range of 1∶200–1∶3,276,800, added to the wells, and incubated for 2 hours at 37°C. Plates were washed six times with PBST, and biotinilated goat anti-mouse IgG, IgM (Sigma), or monoclonal IgG1 or IgG2 (BD Pharmingen) were added to the wells (1 µg/ml) for 1 hour at 37°C. Avidin-HRP (1∶1000; Sigma) was then added and incubation continued for 30 min at 37°C. After six additional washes with PBST, 3,3′,5,5′ tetramethylbenzidine substrate (1-Step TMB; Pierce) was added, and the enzyme reaction proceeded for 20 min. The reaction was stopped with 2 M H_2_SO_4_. The absorbance was quantified in a microplate spectrophotometer at a wavelength of 450 nm. Serum antibody titers were determined by measuring the last dilution that resulted in three standard deviations above background.

### Proliferation Assay

Cultured T-cells were trypsinized, centrifuged at 1000 rpm, and diluted to 1×10^6^ cells/mL in complete RPMI, then labeled with CFSE (Invitrogen). Cells (2×10^5^) were seeded into 96-well plates, incubated with GFP (30 µL, 100 µg/mL), and allowed to proliferate at 37°C for 4 days. FACS was used to assess loss of CFSE in proliferating cells.

### Inflammation Pathology

Four groups (n = 4, all groups) of 8-week old BALB/c mice (Charles River Laboratories) each were injected s.d. in each ear with 10% of the vaccine dosage described previously for PBS, EcJ OMVs, EcN OMVs, and EcN-*LpxM* OMVs. Mice were euthanized 30 hours after injection, and their ears were resected and immediately fixed in 10% formalin. Tissue samples were processed routinely for histopathology and stained with H&E for light microscopy evaluation. Slides were read and graded in a blinded fashion by a licensed pathologist. For gross pathology, a 0–3 system was used: 0, no observable reaction; 1, slight redness only visible by parting fur, no touch sensitivity; 2, redness and swelling with touch sensitivity; 3, extreme inflammation leading to skin cracking/oozing, hair loss, and necrosis. For histological pathology scoring, a 1–5 system was used: 1, no observable change; 2, minor leukocyte recruitment; 3, leukocyte recruitment, apparent vasculature swelling and edema; 4, major leukocyte recruitment, vasculature swelling and edema, loss of structural order in tissue; 5, tissue remodeling and potential necrosis, vasculature rupture and extreme edema, plentiful neutrophils.

### Cytokine Secretion Analyses

For the determination of cytokine secretion, appropriate cells were resuspended at a concentration of 2×10^6^ cells/well in medium RPMI 1640 (supplemented with FCS and antibiotics), seeded into 96-well plates and incubated for 48 h with GFP (5 µg). Cytokine levels of IFN-γ, IL-4, and IL-10 were measured in the supernatants as described above by using standard ELISA kits (eBioscience). For splenic cell analysis, T-cells from pre-boost (t = 4 wks) and post-boost (t = 8 wks) mice were both assessed, but no groups from pre-boost mice showed significant (p<0.05) stimulation; as such, only data from the post-boost mice is discussed below.

### Cytokine Expression Analyses

Cytokine expression analysis was conducted on primary mouse lymphocytes and macrophages. For splenic lymphocytes, cells were stimulated overnight with GFP (5 µg/ml), IL-2 (5 ng/ml) and anti-CD28 (10 µg/ml), and then cultured with brefeldin A. Cells were stained with fluorescent antibodies against the surface markers CD3 (clone 17A2) and CD4 (clone RM4–5), permeabilized, fixed and incubated with antibodies against the cytokines IFN-γ (clone XMG1.2) or IL-10. All incubations were carried out on ice for 30 min. All antibodies were purchased from BD Bioscience or eBioscience. For each sample, at least 50,000 cells were analyzed. The data were collected and analyzed using CELLQuest or FlowJo software and a FACScalibur flow cytometer (Becton Dickinson, San Jose, CA). For macrophages, cells were plated as above and also incubated with increasing concentrations of OMVs. Sixteen hours later, brefeldin A (10 µg/ml) was added and cells were incubated for 6 additional hours. Macrophages were then collected to be stained and analyzed by flow cytometry. Prior to staining, cells were incubated with an anti-Fcγ III/II receptor antibody (BD Biosciences) and 10% normal mouse serum (NMS) in PBS containing BSA (0.1%) and NaN_3_ (0.01%). Cells were stained with antibodies against the surface marker CD11c (clone 223H7), fixed in 2% paraformaldehyde, permeabilized with saponin and then incubated with fluorescent antibodies against the cytokines IL-12 (clone C17.8), IL-10 (clone JES5–16E3) or IL-4 (clone 11B11). Incubations were carried out for 30 min on ice. All antibodies were purchased from BD Biosciences or eBioscience. Data was collected and analyzed as described above.

### Phagocytosis Assay

Bone marrow-derived macrophages were plated in 6-well plates (10^6^ cells/well) for 16 hours and incubated with increasing concentrations of ClyA-GFP(+) EcN or EcJ OMVs. Two hours after the addition of the OMVs, plates were washed vigorously; cells were collected and analyzed by flow cytometry to detect internalized GFP. The data were collected using a FACScalibur flow cytometer and analyzed in CELLQuest software (Becton Dickinson, San Jose, CA). For each sample, at least 30,000 cells were analyzed.

### TLR Stimulation Studies

MyD88 or TLR4 deficient macrophages were cultured and stimulated as described above for 6 hours with OMVs or LPS, and media TNF was then measured by ELISA (Biolegend) according to the manufacturer. For the TLR reconstitution assay, single-TLR-expressing, NF-kB-luciferase(+) HEK293 cells were treated with OMVs or TLR agonist controls, with luciferase activity in lysates measured on a luminometer (Veritas).

### Two-photon Imaging of Lymph Node Tissue

BALB/c mice (n = 3) were injected s.c. between the scapulae with EcN OMVs (100 µg) and/or splenic DCs (1×10^6^ cells), stained with DiO and DiD respectively (Invitrogen), then euthanized after 4 days. After euthanasia, intact draining cervical lymph nodes were resected, placed in PBS, and immediately imaged via two-photon excited fluorescence microscopy on a custom setup. Imaging was conducted on a custom-designed microscope using a train of 830-nm, 90-MHz, 140-fs pulses from a Ti:sapphire laser oscillator (Chameleon; Coherent) for excitation. Laser scanning and data acquisition were controlled by ScanImage software [34]. For high-resolution imaging of labeled cells and particles respectively a 20X (numerical aperture: 1.0) water-immersion objective (Zeiss; W Plan-APO Chromat). Second harmonic generation from the extracellular matrix in lymph node was detected using a 417-nm centered wavelength filter. Fluorescence was detected using emission filters with 510-nm and 641-nm center wavelength with 65-nm bandwidth to image. Images shown are 20 µm stacks that were compiled using a maximum projection and a median filter was applied using Image J software (http://imagej.nih.gov/ij/).

### T-cell Activation Analyses

T-cell activation by stimulated DCs was assayed as described previously [35]–[36]. Briefly, 96-well plates were incubated overnight with anti-mouse CD3 (10 µg/mL, clone 145–2C11; BD Biosciences) at 4 C, after which plates were washed 3 times with complete RPMI. T cells were then labeled with CFSE as described and seeded into wells at 2×10^5^ cell/well. T-cells were co-incubated with DCs (4×10^4^ cell/well), anti-mouse CD28 (5 µg/mL, clone 37.51, Biolegend), and a range (1 to 100 µg/mL) EcJ or EcN OMVs. Samples prepared for flow cytometry analysis were incubated for 48 h at 37°C, then incubated for an additional 4–6 h with 25 ng/mL PMA, 1 µg/mL ionomycin, and 10 µg/mL brefeldin A. Samples prepared for proliferation analysis were incubated at 37 C for 7 days, then trypsinized and analyzed by flow cytometry for loss of CFSE staining.

### Statistical Analyses

Statistical significance was determined by unpaired two-tailed Student’s t-test or by ANOVA grouped test with supplemental Tukey’s HSD tests when appropriate. Significance was determined to be at a confidence interval of P<0.05. Proteomic data was cross-referenced between three *E. coli* proteome libraries to determine identifications at P<0.001.

## Results

### EcN OMVs are engineered as recombinant antigen carriers via a ClyA-fusion mechanism

**Figure 1:**
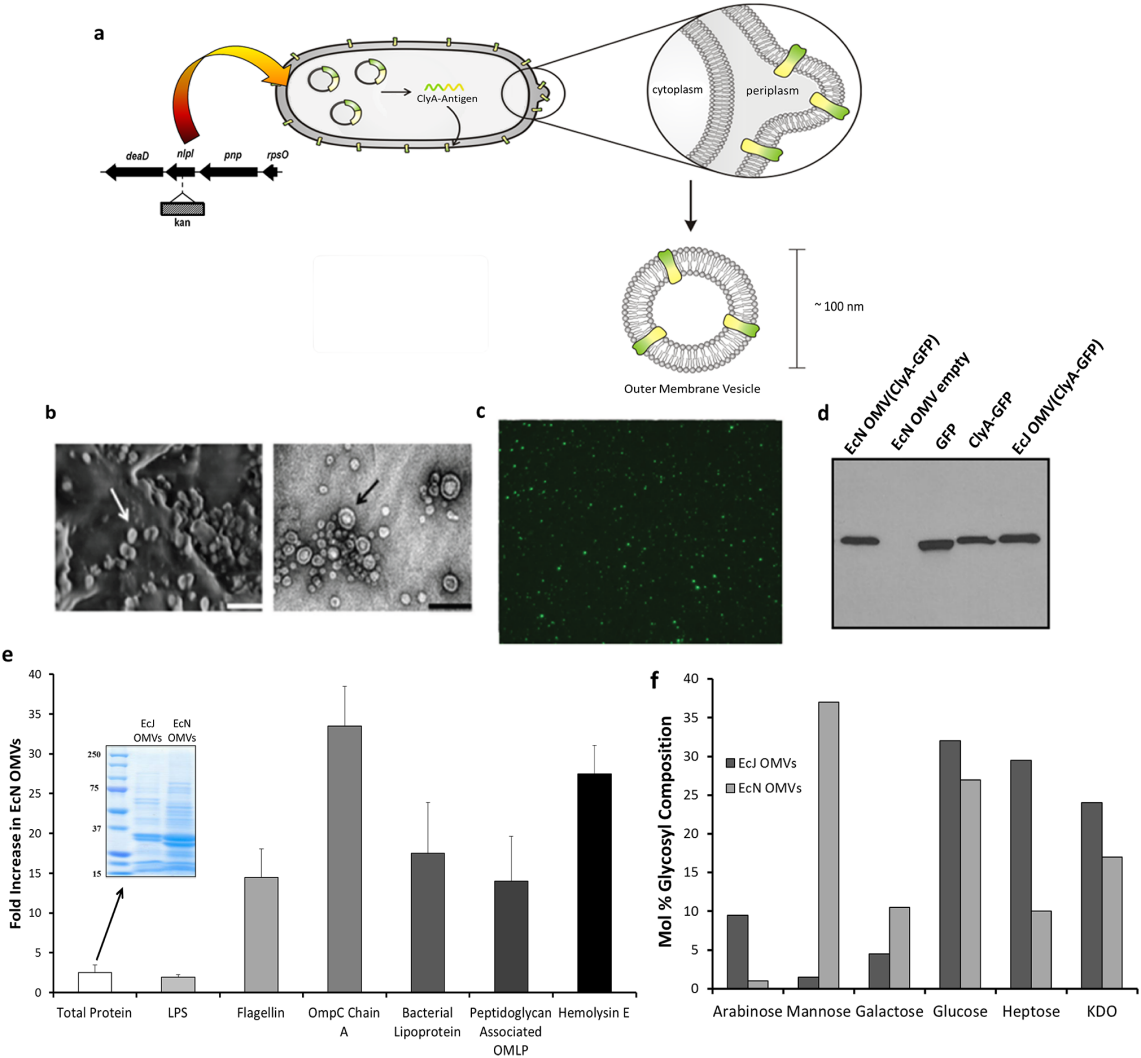
EcN *nlpI* mutant strain produces functional, recombinant subunit antigen carriers. *a,* Schematic representing the generation of engineered, custom antigen-presenting OMVs from *nlpI*-mutant, hypervesiculating *E. coli*. *b,* Representative FE-SEM micrographs (left panel) demonstrate predominantly uniform size and shape of EcN OMVs, while TEM micrographs (right panel) highlight characteristic bilipid membrane (OMVs indicated by arrows; scale bars = 200 nm). *c,* Representative fluorescent micrograph (100x objective) of purified OMVs from EcN cultures transformed with a ClyA-GFP plasmid vector, demonstrating maintained folding of displayed antigen. *d,* Western blot with anti-GFP antibodies of OMV suspensions from EcN and EcJ cultures expressing ClyA-GFP. *e,f* Enrichment of immunologically relevant proteins *(* ***e*** *)* and sugars *(f)* in EcN OMVs relative to EcJ OMVs (ClyA-antigen-normalized samples), characterized by chromatography-assisted mass spectrometry. *e,* Insert: Coomasie-stained gel showing total protein of antigen-normalized samples. Error bars represent mean + SD of biological replicates.

Previously, we reported the successful expression and functional folding of a variety of recombinant antigens on the surface of K12 *E. coli* OMVs by inducing expression of a chimera consisting of the antigen fused to the C-terminus of enterobacterial cytotoxin ClyA [37]. For the present study, green fluorescent protein (GFP) was selected as the primary model antigen for two reasons. First, GFP’s natural fluorescence allowed simple quantitation of antigen content in all vaccine preparations, owing to the ClyA-antigen chimera’s capacity to retain proper antigen folding upon outer membrane insertion [29]. GFP’s second advantage was its poor immunogenicity in mice, which allowed for maximal demonstration of the vaccine carrier’s immunostimulatory potential. OMV overproduction was induced in EcN via genetic knockout of *nlpI* [37]–[38] to produce the antigen delivery platform (Fig. 1a). OMVs released by the hypervesiculating EcN mutants expressing ClyA-GFP exhibited uniform size and shape while retaining characteristic bilipid membrane structure (Fig. 1b) and carried fluorescent membrane-bound GFP (Fig. 1c). To demonstrate the effect of probiotic strain-selection on the immune response, similar GFP-containing OMVs (Fig. S2a–b in File S1) were produced as a control using the non-probiotic K12 *E. coli* strain JC8031 (abbreviated EcJ) [29], [37]. Western blot analysis confirmed comparable ClyA-GFP content in both OMV preparations (Fig. 1d), allowing for meaningful comparisons between the two strains’ abilities to trigger an immune response against the model antigen. To begin to better understand the molecular basis for the observed immune responses, we additionally investigated LPS, proteomic, and glycosyl differences between the EcJ and EcN OMV TLR-agonist composition. EcN and EcJ OMVs contained similar amounts of membrane-bound LPS (Fig. 1e), a known key contributor in OMV-induced immune responses [12]. In stark contrast, several major PAMP-rich peptide TLR agonists were substantially enriched in EcN OMVs, particularly the potent TLR5 agonist flagellin. Glycosyl analysis of sugar composition (Fig. 1f) revealed substantial enrichment of mannose in the EcN OMVs, an agonist of the mannose-binding lectin pathway. These compositional differences may form the molecular basis of the unique T_H_1-biased immune response imparted by EcN OMVs that is described in the following section.

### Immunization with EcN OMVs induces antigen-specific T_H_1-biased immunity

**Figure 2:**
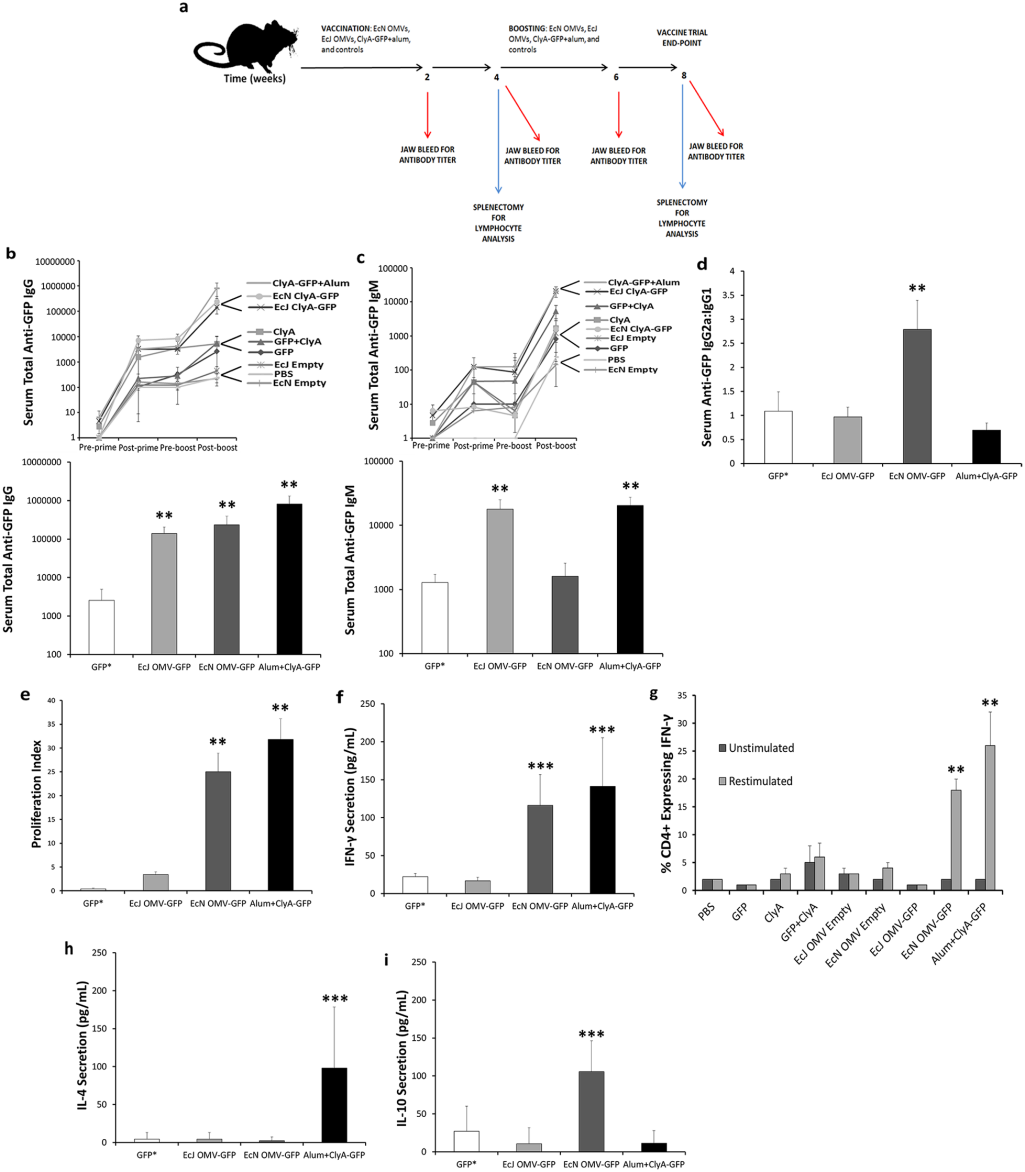
EcN OMV antigen carriers generate robust, T_H_1-biased antigen-specific humoral and cellular immunity in a mouse model. *a,* Vaccination protocol for immunization of BALB/c mice against a model antigen. *b,c,* Time-evolution (upper panel) and terminal titers (lower panel) of antigen-specific IgG *(b)* and IgM *(c)* from BALB/c mice vaccinated (primed) and boosted once with antigen-normalized doses (n = 5, each group). Experimental groups indicated are as follows: mice injected with a mixture of recombinant ClyA-GFP and alum, ClyA-GFP+Alum; with EcN OMVs displaying ClyA-GFP, EcN ClyA-GFP; with EcJ OMVs displaying ClyA-GFP, EcJ ClyA-GFP; with recombinant ClyA in PBS alone, ClyA; with a mixture of ClyA and GFP in PBS, GFP+ClyA; with recombinant GFP in PBS alone, GFP; with EcN OMVs not displaying any recombinant ClyA-antigen fusion, EcN Empty; with EcJ OMVs not displaying any recombinant ClyA-antigen fusion, EcJ Empty; and with PBS alone, PBS. *d,* Ratio of IgG2a:IgG1 antibodies from terminal total IgG titers in *b. e-i,* CFSE-measured proliferation index *(e)*, IFN- γ secretion *(f)* and expression *(g)*, IL-4 secretion *(h)*, and IL-10 secretion *(i)* from cultured, GFP-restimulated splenic T-cells harvested from end-point subjects used in *b-d* (n = 5, each group). GFP* data are representative of GFP, ClyA, GFP+ClyA, EcJ Empty and EcN Empty, and PBS control groups. **P<0.001, ***P<0.05. All values are given as mean + or +/− SD.

To test the ability of EcN OMVs to generate a strong adaptive response to GFP, we immunized BALB/c mice via subcutaneous injection with formulations of OMVs containing ClyA-GFP and free of additional adjuvant (Fig. 2a). To ensure the presence of a substantially competitive positive control, we separately injected vesicle-free ClyA-GFP adsorbed onto the aluminum hydroxide (alum) adjuvant delivery system, Alhydrogel. IgG titers assayed four weeks after the final boost indicated induction of a strong humoral response by both EcN and EcJ OMVs comparable to the gold standard of alum (Fig. 2b), consistent with previous work [29] and reflective of progressive generation of a robust response. However, IgM titers showed some divergence in the humoral responses (Fig. 2c), with lower IgM levels generated by EcN OMVs indicating either early class-switching or a discrepancy in B-leukocyte stimulation by membranous endotoxins. Further divergence could be seen in IgG1 versus IgG2a titers (Fig. 2d). The EcN OMVs elicited an IgG2a-dominant humoral response, which taken together with the relative decrease in final IgM titer suggests induction of a T_H_1-facilitated immune response consistent with heightened cellular immunity stimulation [39]. In contrast, EcJ OMVs mirrored the alum-positive control with a larger IgG1 fraction, suggesting a response more in line with standard adjuvants. As a result, these data not only indicate that the EcN OMVs can function as an effective antigen carrier for stimulating humoral immunity, but additionally suggest a strain-dependent advantage conferred by EcN in stimulating a traditionally elusive T_H_1 response.

Further analysis of the mice’s OMV-induced adaptive immunity revealed a favorable T-cell response reflective of the EcN OMVs’ unique humoral response. Specifically, spleen-derived T-cells from post-boost mice vaccinated with ClyA-GFP EcN OMVs had proliferation responses to antigen restimulation comparable to the alum-positive ClyA-GFP control and significantly greater than that triggered by EcJ OMVs (Fig. 2e). This result is generally indicative of a significant immunostimulatory effect on naïve T-cell populations, and is in direct agreement with the driving hypothesis that the EcN membrane may be privileged as an adjuvant material in its ability to actively target T cells. Moreover, EcN but not EcJ OMVs promoted a similarly dramatic increase in antigen-restimulated T-cell IFN-γ secretion (Fig. 2f) and expression levels (Fig. 2g). Taken together, these results highlight the ability of EcN OMVs to induce a strong cellular immune response, which correlates well with the T_H_1 response suggested by the IgG2a:IgG1 ratio. Depressed IL-4 levels (Fig. 2h) relative to IFN-γ further support the induction of a favorable T_H_1-biased response by EcN OMVs. This level of T_H_1-indicative IFN- γ/IL-4 discrepancy has rarely been reported in the literature for adjuvant-free subunuit vaccine carriers, making it of particular interest in this study. Importantly, this also sets the EcN OMV carrier apart from standard adjuvants such as alum, which produce a mixed T_H_1/T_H_2 (T_H_2 dominant) response that is often insufficient at leveraging the advantage of T_H_1 immunity against certain intracellular pathogens. Also of note was the observation that IL-10 secretory activity from EcN OMV restimulation was elevated relative to EcJ OMVs and the alum-positive control (Fig. 2i), despite the overall T-cell population participating in IL-10 secretion not being as dramatically elevated. Considering the successful induction of elevated T-cell IFN-γ levels, this suggests a unique (though possibly minor) use of IL-10 in a supplementary and indirectly beneficial role in generating a favorable and non-detrimental T-cell response [40], though further study is required to determine if this might have a practical role in modulating either adaptive immunity or associated inflammatory responses (discussed below). Taken together, the antigen-specific humoral and cellular immunity induced by the EcN OMV carriers are indicative both of a protective vaccine response [41] and, more importantly, a response tuned toward potential use against pathogens that are most effectively neutralized by the more elusive T_H_1-biased response [42].

### EcN OMVs engage both LPS-dependent and independent innate immunity

**Figure 3:**
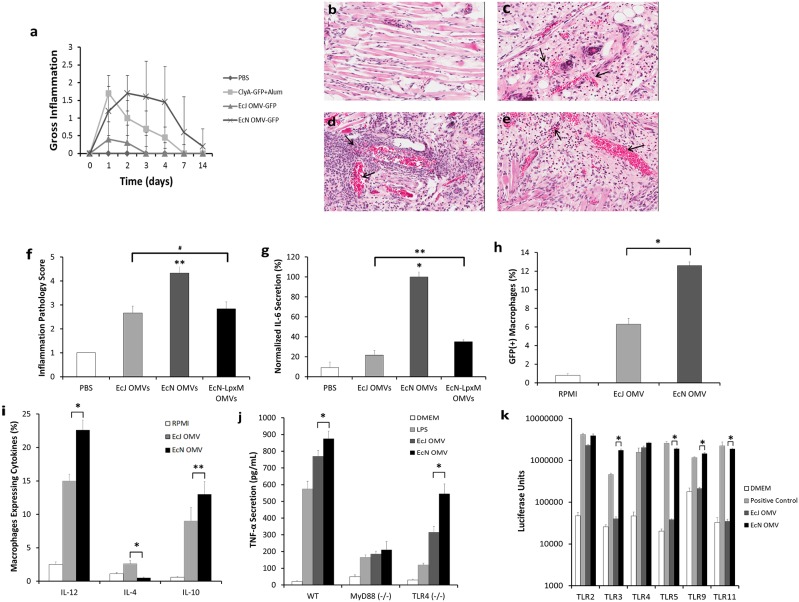
EcN OMV carriers are capable of potent self-adjuvanting engagement of innate immunity. *a,* Observed macroscopic inflammation (scored 0–3 for severity) at s.c. injection site (n = 5, each group). *B–e,* Representative dermal histological sections from the injection site (t = 30 h) in BALB/c mice (n = 4, each group), injected with PBS *(b)*, EcJ OMVs (**c**), EcN OMVs *(d)*, or *lpxM*-mutant EcN OMVs *(e)*. Arrows indicate vasculature swelling and leukocyte recruitment, which is substantially enhanced by EcN OMVs and reduced, but not eliminated, by the *lpxM* mutation. *f,* Inflammation pathology scoring of *b–e* on a 0–5 scale. *g,* IL-6 secretion levels, normalized to the highest value in the data set, from primary mouse bone-marrow derived macrophages incubated with OMVs used in *b-e. h,i,* Flow cytometry *(h)* and cytokine secretion analysis *(i)* of primary mouse bone-marrow derived macrophages incubated with EcJ and EcN OMVs (n = 3, each group), demonstrating enhanced macrophage stimulation by EcN OMVs. *j,* TNF-α dependent activation of wild-type (WT), MyD88^−/−^, and TLR4^−/−^ mouse macrophages incubated with EcJ and EcN OMVs (n = 5, each group) reveal MyD88-dependent, TLR4-dependent and TLR4-independent stimulation via EcN OMVs. *k,* TLR activation in single-TLR expressing human embryonic kidney (HEK) cells incubated with EcJ and EcN OMVs (n = 3, each group), demonstrating broad TLR activation by EcN OMVs relative to exclusively TLR2/4 activation by EcJ OMVs. *P<0.001, **P<0.05, ^#^No statistically significant difference. All values are given as mean + or +/− SD.

A key determining factor in adaptive immune response generation by a vaccine is the extent to which innate immunity is engaged [2]. While on a macroscopic level OMV vaccine inflammation was observed to be similar to that of the alum control in duration and recovery time (Fig. 3a), such observations are not necessarily reflective of an adjuvant’s capacity for local immune cell engagement and recruitment. Hence, we further explored the capacity of EcN OMVs to robustly induce an innate response. Using a subdermal ear injection model in BALB/c mice, and subsequent histological analysis, we assessed the acute inflammatory response generated by both EcN and EcJ OMVs. The resulting inflammopathology revealed surprisingly marked differences. At 30 h post-injection, EcN OMVs dramatically remodeled the dermal tissue, caused local vasculature swelling, and recruited dense populations of leukocytes (Fig. 3d) relative to a blank PBS control (Fig. 3b). Such a reaction is an established sign of an adjuvanting material capable of stimulating the required innate immunity activation for a good vaccine response [2], [43]. EcJ OMVs, on the other hand, stimulated a much less dramatic inflammatory response with an equivalent OMV dosage (Fig. 3c). Taken together, these data imply that the probiotic strain-dependent nature of EcN OMVs’ unique immune response is in part dependent on a strong stimulation of innate immunity.

Importantly, the different innate immune stimulation from the EcN and EcJ strains resulted from equivalent doses of OMV-bound LPS, generally considered to be the most important innate bacterial immunomodulator [12]. This result implies that the immunostimulatory mechanism for the EcN OMVs is not fully dependent on the LPS. Therefore, we next investigated whether or not direct reduction of LPS-induced endotoxicity would substantially attenuate the enhanced innate response observed with EcN OMVs. A genetic knockout of the *lpxM* gene in *E. coli* inactivates the msbB lipid A acyltransferase and is known to minimize LPS-based endotoxicity [44]; therefore, *lpxM* was knocked out in the EcN *nlpl* mutant to generate mutant EcN OMVs (Fig. S2c–d in File S1) and allow investigation of the influence of LPS on the activation of the innate response. Following identical subdermal mouse ear injections, the previously observed inflammopathology was partially attenuated but ultimately still reflective of an appreciable inflammatory response (Fig. 3e), both on the tissue (Fig. 3f) and cellular (Fig. 3g) levels. Follow-up studies of vaccine efficacy in BALB/c mice under identical conditions as those described above additionally demonstrated that the *lpxM* mutation did not eliminate the ability of EcN OMVs to stimulate strong anti-GFP humoral immunity (Fig. S3 in File S1). As a result, it would appear that a non-endotoxicity-dependent, strain-dependent advantage in innate immunity stimulation is conferred by using EcN as an OMV source, possibly due to the presence of other highly active immunostimulatory membranous PAMPs [45] and TLR agonists, as suggested by the proteomic analysis discussed above.

Analysis of EcN OMV macrophage stimulation, an important cellular component of innate immunity, provided additional support for an enhanced mechanism of innate immunity stimulation provided by the EcN source. When bone marrow-derived mouse macrophages (BMMs) from wild-type BALB/c mice were incubated with equivalent amounts of ClyA-GFP-containing EcN and EcJ OMVs, they internalized a significantly greater number of the former at a variety of OMV concentrations (Fig. 3h). These results suggest that the EcN outer membrane is a more potent activator of macrophage phagocytosis. Further analysis of BMM cytokine expression profiles following OMV incubation additionally revealed a significant discrepancy in IL-12 and IL-4 expression induced by EcN and EcJ OMVs (Fig. 3i). Specifically, EcN OMVs stimulated elevated IL-12 but not IL-4 expression relative to EcJ OMVs, indicating a propensity for EcN OMVs to facilitate T_H_1 dominance during the adaptive immunity transition. In addition, the elevated IL-10 levels likely indicate a more potent general stimulation of phagocytic activity on the part of the EcN OMVs [46]. This may highlight a synergistic coupling of enhanced targeting via superior PAMP-dependent TLR crosslinking and subsequent intracellular delivery of membranous and soluble bacterial factors [12]. The opposite expression levels induced by EcJ OMVs in turn suggests facilitation of a mechanism more in line with T_H_2 bias [47], though without additional studies this facet of the response is primarily useful as in highlighting a general discrepancy in immunity bias as being crucial to the mechanistic benefits conferred by using the EcN OMVs.

Past studies of wild-type EcN bacteria suggest that its immunomodulatory capacity is derived from TLR-dependent stimulation of antigen-presenting cells and T-cells that makes use of a variety of enriched TLR agonists [48]. Building on the above findings concerning innate immunity stimulation, macrophages were selected to determine a potential connection between TLR pathway stimulation and enhanced EcN OMV adjuvancy. First, we confirmed the presence of both TLR4-dependent and TLR4-independent stimulation in the EcN OMVs’ adjuvancy using MyD88 and TLR4 macrophage knockouts (Fig. 3j). Further testing with cell lines genetically restricted to single-TLR expression was then used to characterize the implied broadened stimulation spectrum unique to the EcN OMVs (Fig. 3k). Uniquely strong stimulation of TLR2, TLR3, TLR4, TLR5, TLR9, and TLR11 were all seen with observed via the EcN OMVs. TLR2/4 stimulation is consistent with *E. coli* bacterial membranes, while TLR5 and TLR9 stimulation (likely via enriched EcN membrane components such as flagellin) were also expected due to previous evidence of retained EcN-derived immunostimulation [17], [19], [20]. The TLR3 expression is more challenging to immediately understand without further study, and could have resulted either from intracellular delivery of some unknown bacterial factor or possibly cross-activation via residual TLR5 pathway expression. Regardless, these two experiments together revealed that TLR2/4-dependent, TLR5-dependent, and non-TLR2/4/5 MyD88-dependent mechanisms had all been harnessed by EcN OMVs. These results correlate well with past *in vitro* mechanistic studies on intact EcN bacteria [19], [20], [48], and support the premise that the EcN OMVs’ T_H_1-biasing capacity is derived from the acellular isolation of EcN’s unique probiotic immunity.

### Adaptive immunity stimulation is facilitated by APC targeting and T_H_1-biased activation

**Figure 4:**
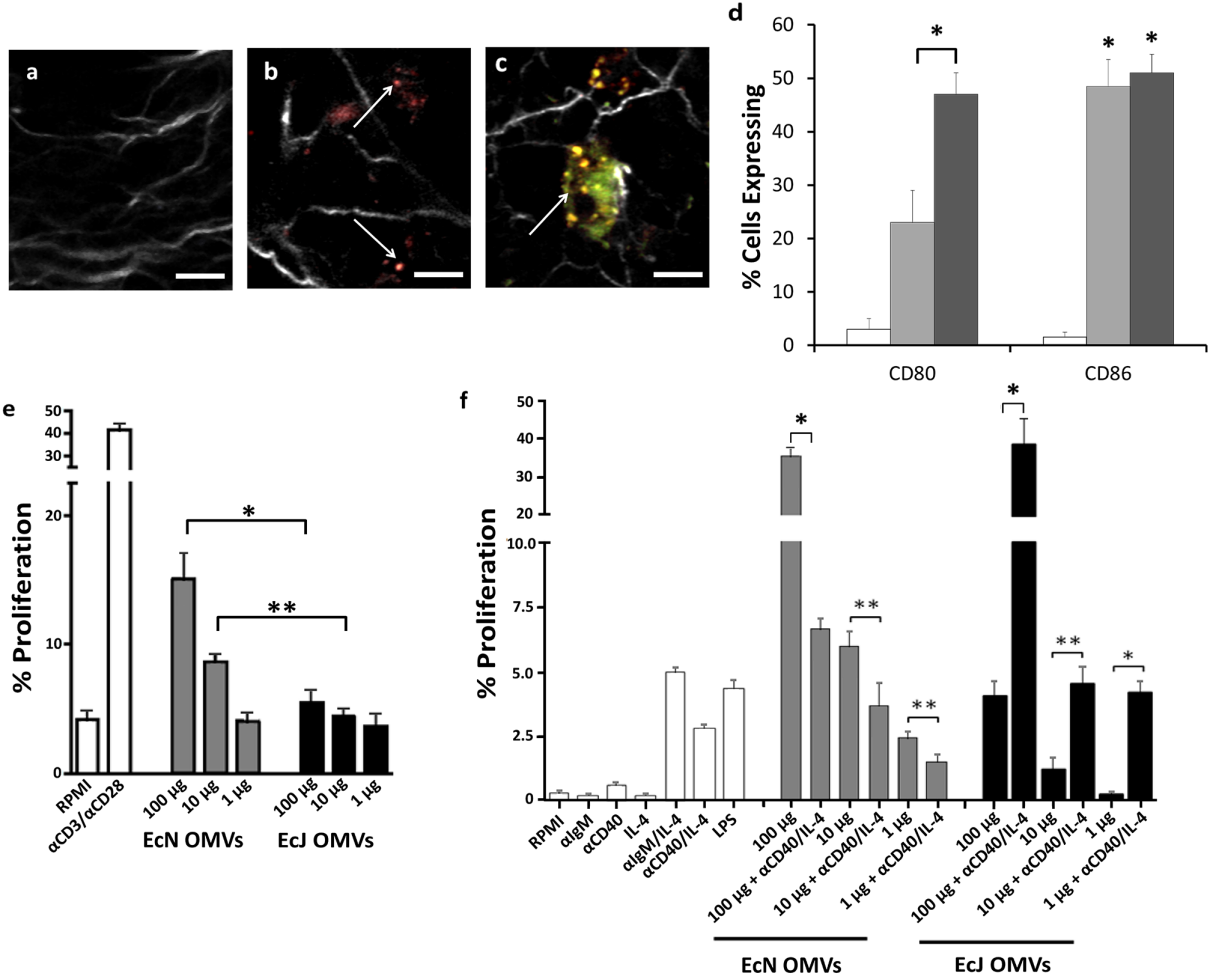
EcN OMV carriers effectively stimulate adaptive immune cells. *a–c,* Representative two-photon microscopy of draining lymph nodes from mice (n = 3, all groups) injected with PBS *(a)*, fluorescently stained EcN OMVs *(b)*, and fluorescently stained EcN OMVs and splenic dendritic cells (DCs) *(c)*. White = collagen, red = OMVs, green = DCs, and yellow = OMV/DC overlap. Arrows denote OMVs *(b)* or DCs *(c)*. Scale bar = 15 µm. *d,* Flow cytometry analysis of primary mouse splenic DC stimulation by EcJ and EcN OMVs (n = 3, all groups), identifying subpopulations displaying surface markers for activation CD80 and CD86. *e,* Proliferation of primary mouse splenic T-cells following coculture with DCs from *d* (n = 3, all groups) demonstrates more potent activation via EcN OMVs. αCD3/αCD26 = positive control. *f,* Proliferation of primary mouse bone marrow-derived B-cells following incubation with OMVs, with or without additional T-cell helper factors (n = 3, each group). Anti-IgM/IL-4, anti-CD40/IL-4, and LPS = positive controls. *P<0.01, **P<0.05. All values are given as mean + SD.

An important link between a heightened inflammatory response and the development of a strong adaptive T-cell response are antigen-loaded dendritic cells (DCs) that migrate from the site of inflammation to local lymph nodes [9]. Two-photon microscopy of extracted lymph nodes was used to confirm that OMVs drained into lymph nodes following subcutaneous injection, where they spatially overlapped with adoptively transferred dendritic cells (DCs) (Fig. 4a–c). These results confirm that the OMV carriers and their delivered antigens are available to lymphoid-localized DCs, T-cells, and B-cells [49]. Subsequent *in vitro* stimulation of separately purified murine splenic DCs revealed that while both EcN and EcJ OMVs substantially upregulated CD86, EcN OMVs had a greater capacity to additionally upregulate CD80 as well (Fig. 4d). This is promising, as CD80 has been implicated in modulating regulatory immunity in addition to traditional stimulatory cellular immunity [50].

Similar studies using murine DC and naïve splenic T-cell coculture (Fig. 4e) demonstrated stronger DC-facilitated T-cell stimulation by EcN OMVs as well. Specifically, the pronounced increase in naïve T-cell activation by BMDCs primed with a variety of EcN OMV concentrations relative to those primed with EcJ OMVs suggests that the selection of EcN as the OMV source improved the ability of the OMVs to effectively interface with and stimulate APCs. Additionally, the ability of the EcJ OMV control to also stimulate T-cells through BMDC-dependent activation, albeit to a lesser extent than EcN OMVs, helps rule out the possibility that they caused a DC-dependent T-cell suppression [17], which would have made conclusions concerning a strain-dependent T_H_1/T_H_2-bias discrepancy difficult.

Taken together, the enhanced stimulation of both macrophages and DCs by EcN OMVs builds a strong case for a PAMP-dependent mechanism for enhanced activation of professional APCs as a crucial component of the robust induction of adaptive immunity to a recombinant antigen. These data led us to consider that the EcN OMVs’ immunomodulatory capability might take advantage of similarly improved interactions with the third major APC, namely B-cells. Accordingly, we measured induced proliferation of splenic mouse B-cells via incubation with EcN and EcJ OMVs. This analysis revealed that EcN OMVs possessed significant potential for type 1 T-cell independent B-cell activation due to their ability to stimulate B-cell proliferation in the absence of T-cell helper factors at a variety of OMV concentrations (Fig. 4f). Such activation is not uncommon in pathogen infection [51], and likely resulted from a combination of the presence of EcN PAMPs and OMV nanoscale avidity enhancement [5], [52], possibly facilitated by the elevated TLR9 stimulation noted previously. Interestingly, this phenomenon was in strong contrast to the EcJ OMV control’s ability to activate B-cells, which was entirely dependent on the addition of T-cell helper factors IL-4 and anti-CD40. Such a strong discrepancy further highlights the important benefit EcN provides to *E. coli* OMV antigen carriers, potentially providing robust humoral immunity through a supplemental T-cell independent mechanism that can work in parallel to, and perhaps even uniquely enhance, the establishment of a T_H_1-biased adaptive response.

## Discussion

While being an interest of the immunological community for decades, probiotic bacteria remain something of an enigma when it comes to medical applications. Utility of their potent immunomodulatory capacity has been reserved primarily for nutritional supplement-based approaches to managing clinical conditions such as irritable bowel disease and general allergy [17]. Research into their mechanism of action has been on the rise in recent years, however, and the emerging picture concerning their capacity for locally [19], [20] and systemically [17] altering the immune system indicates the ability to strike a non-trivial balance between actively targeting and antagonizing key immune cells while simultaneously suppressing components that would exacerbate an active response. Such complexity is often the hallmark of organisms that have evolved to have an intricate and potent interaction with the host immune system, and it is this complexity that has made probiotic bacteria simultaneously attractive to study and difficult to apply in the medical field.

In this study, we assessed the efficacy and mechanism of OMVs to isolate the PAMP-driven immunostimulatory nature of probiotic bacteria for recombinant subunit vaccine delivery applications. OMVs are bacterially-derived, nanoscale proteoliposomes naturally enriched with immunoactive components ranging from LPS, to and various bacterial lipoproteins, to lumen-sequestered “vita-PAMPs”, creating intriguing pathogen-like particles – that is, particles the immune system can be tricked into believing are, for all practical intents and purposes, active pathogens. OMVs have the unique ability to combine the mechanistic advantages of a specific pathogen mimic as a delivery vector with the intrinsic advantages of a nanoscale delivery vector, such as enhanced cellular uptake through ligand-dependent surface receptor cross-linking [53]. By using a ClyA fusion-chimera approach to exogenous antigen production, we were able to create a vaccine carrier particle with surface displayed antigen that could be produced as a single-component, self-adjuvanting entity in simple bacterial culture that can take full advantage of the attractive vaccine carrier features of the bacterial OMV.

Collectively, our findings indicate that EcN OMVs generate a relatively rare adaptive response to a recombinant antigen that current adjuvant technology fails to consistently and/or simplistically achieve: a functional anti-antigen humoral response more than 100-fold stronger than antigen alone, indicative of a response strong enough to confer immunity [41], coupled with a T-cell response whose strength and T_H_1 specificity suggest the conferral of cellular immunity on a level that would substantially enhance efficacy against intracellular pathogens [42]. These characteristics suggest that EcN OMV vaccine carriers can potentially fill an important vaccine niche and address the critical unmet clinical need of a practical and widely applicable platform for creating vaccines against a variety of high-profile intracellular pathogens. Moreover, probing the way these OMVs directly interface on a molecular and cellular level with both innate and adaptive immunity revealed a unique capacity for robust and multi-faceted stimulation that is dependent not only on the nature of bacterial OMVs in general, but specifically reflects their ability to harness specific advantages of the EcN bacteria and couple them with systemic trafficking and cellular targeting. Isolating the targeted adjuvant component of a highly immunoactive, yet immunosuppressive bacterial strain has led to an antigen delivery system capable of robust and complete innate and adaptive immune stimulation.

Of note is the apparent discrepancy that emerges in this study between an immune reaction to an OMV in its entirety (and, correspondingly, its source strain) and a specific delivered recombinant subunit antigen. Recent work by Kim et al. [18] indicated that standard K-12 *E. coli* OMVs induce a T_H_1-biased immune response that was shown to be essential for protection against *E. coli*-induced lethality post-challenge in a similarly vaccinated murine model. Our study therefore indicates an intriguing dichotomy: bacterial OMVs apparently have an intrinsic capacity to induce T_H_1 bias, but may lack the capacity to necessarily direct it against specific protein-based antigens. In our study, it was clear that the EcJ OMVs lacked sufficiently directed T_H_1-biased immunostimulation to couple such immunity to the recombinant ClyA-GFP subunit antigen. While *in vitro* analysis showed only moderate enhancement of macrophage and T-cell activation via EcN OMVs, the B-cell stimulation data indicates a clearer discrepancy between the two. It is possible, therefore, that the mechanism of antigen-directed immunity enhancement by the EcN OMVs diverges from more standard *E. coli* OMVs to present recombinant subunit antigens that directly engage with key immune cells, which is in keeping with our mechanistic hypothesis. We are currently working to better elucidate the cause behind these differences, which, given the extent of the discrepancy between both innate and adaptive immune responses, could very well extend beyond a singular T_H_1 bias to more complex responses incorporating a T_H_17 bias as well [18].

To engineer and optimize bacterial OMVs as a vaccine delivery carrier, we had a specific interest in demonstrating how using recombinant subunit antigens, rather than whole pathogens need not sacrifice the immunological advantages of holistic pathogen-host responses. OMVs as exogenous antigen carriers have been previously established, by our group [29] and others [16], as a promising vaccine delivery technology. Our goal in this paper, therefore, was to establish the capacity of rationally engineered OMV-based carriers to bridge the gap between some of the more complex advantages still retained exclusively by whole-pathogen vaccine approaches and biomolecular nanocarriers. The capacity of engineered EcN OMVs to bias recombinant subunit antigen-specific immunity strongly towards a T_H_1 response is a core discovery of this study, but it is only one of several advantages illuminated. For example, the vaccine carrier’s observed induction of robust humoral, T_H_1-indicative immunity is particularly meaningful when considering the antigens were additionally presented to the immune system as functionally folded “surface antigens” on the adjuvanting OMV particle. Unlike some of the recently developed adjuvant system alternatives designed to enhance T_H_1-type immunity induction [2], [8], the EcN-derived OMVs have a unique capability to generate recognition immunity against a full range of linear epitopes (via MHC-facilitated T-cell receptor presentation) as well as conformational epitopes (via direct surface presentation to B-cell receptors). In the past, the field had been forced to write-off such an advantage as a necessary loss to move beyond working with whole pathogens. As the EcN OMV our platform is specifically derived from the very biomolecular material responsible for natural and diverse antigen presentation by bacteria, theadvantage is instead preserved and enhanced beyond traditional gold standard adjuvants.

Moreover, this approach to vaccine carrier engineering does not merely preserve advantages of working with whole pathogens – it additionally provides avenues for rational tuning and optimization of antigen presentation as well as the targeted immune response itself. By demonstrating the immune response to a well-characterized model exogenous protein, GFP, we’ve demonstrated that simple protein engineering can lead to the successful coupling of a bacterially stimulated immune response with a non-bacterial antigen. Additionally, our findings with the LPS endotoxicity mutation indicates an opportunity through biomolecular engineering to tune dependence on endotoxicity to a minimal level that reduces the vaccine’s side effects without sacrificing the innate immunostimulatory advantage of probiotic strain-derived OMVs, giving the platform an impactful advantage over other bacterially-derived technologies. Such flexibility is of note with respect to the practical implementation of this technology. Even though fairly large quantities of OMVs beyond our demonstrated vaccine doses have been required in past studies to trigger detrimental responses in murine models [54], [55], the ability to address such a concern in a targeted fashion is essential for bacterially-derived vaccine carriers. Moreover, additional avenues of rational, multivalent modification – such as polyvalent antigen cocktail inclusion [56], pathogen-targeted glycosylation [57], and even particle size modification [53] through additional genetic deregulation of vesiculation control – further contribute to the diverse potential of this platform to facilitate vaccine engineering’s transition from simple stimulatory carrier particles to utilizing more advanced and holistic molecular mimicry of pathogen identity while retaining the feasibility of its utility.

The study presented here builds a case for the efficacy of a bacterially-derived, pathogen-like particle vaccine delivery carrier for a broad range of vaccine applications. Harnessing the potent and broadly integrated immunostimulation of probiotic bacteria and merging it with the widely-used capacity of bacteria to recombinantly express exogenous antigens from almost every classification of vaccine target, has led to a vaccine delivery platform that requires only the expression of an antigenic gene of interest to generate vaccines with potentially far-reaching immunological and clinical impact. In this way, we anticipate that the unique combination of non-infectious immunomodulation and a capacity for diverse, rational molecular engineering – without sacrificing simple and scalable production capabilities – can provide a template for the development of potent new vaccines derived from previously overlooked biological sources.

## Supporting Information

File S1 **Supporting figures.** Figure S1, Isolation of EcN immunostimulatory factors from immunosuppressive ones via OMV formation leads to an induction of a strong immune response. (A) Natural immunosuppressive function of Nissle 1917 *E. coli* (EcN), focusing on direct effector action on αβ T-lymphocytes. (B) By isolating the targeting and immunostimulatory potential of the EcN membrane from EcN’s secretory immunosuppressive capabilities, and turning it into a bionanoparticle delivery device for the natural immunostimulatory milieu present in bacterial OMVs already, the normal immunosuppressive function of the probiotic bacteria is replaced with a powerful adjuvanting effect. Figure S2, Further comparative analysis of EcN and EcJ OMV vaccine formulations. (A) Dynamic light scattering hydrodynamic z-average particle sizes of EcJ and EcN OMVs (formulations assessed in PBS). (B) OMV zeta potentials assessed in PBS. (C) Dynamic light scattering hydrodynamic z-average particle sizes of EcN and EcN-*lpxM* OMVs (formulations assessed in PBS). (D) GFP fluorescence-standardized ClyA-GFP(+) vaccine doses of EcN and EcN-*lpxM* OMVs assayed for total protein content via BCA assay. ^#^No significant difference (P>0.05). All values are given as mean +/− SD. Figure S3, EcN *lpxm* mutation does not result in detrimental loss of robust humoral immunity stimulation in a mouse model. Terminal titers of antigen-specific IgG from BALB/c mice vaccinated (primed) and boosted once with antigen-normalized doses (n = 5, each group). Experimental groups indicated are as follows: mice injected with recombinant GFP in PBS alone, GFP; with EcN OMVs from ECN containing the *lpxM* mutation, displaying ClyA-GFP, EcN-LpxM OMV-GFP; with EcN OMVs from non-lpxM mutant EcN, displaying ClyA-GFP, EcN OMV-GFP; with a mixture of recombinant ClyA-GFP and alum, Alum+ClyA-GFP. **P<0.001.
(DOCX)
