# Supplementary material for: Mechanistic Insight into the TH1-Biased Immune Response to Recombinant Subunit Vaccines Delivered by Probiotic Bacteria-Derived Outer Membrane Vesicles
Source: PLoS One. 2014 Nov 26;9(11):e112802. doi: 10.1371/journal.pone.0112802 (PMC4245113; doi:10.1371/journal.pone.0112802)
Supplement: File S1 — Supporting figures. Figure S1, Isolation of EcN immunostimulatory factors from immunosuppressive ones via OMV formation leads to an induction of a strong immune response. (A) Natural immunosuppressive function of Nissle 1917 E. coli (EcN), focusing on direct effector action on αβ T-lymphocytes. (B) By isolating the targeting and immunostimulatory potential of the EcN membrane from EcN’s secretory immunosuppressive capabilities, and turning it into a bionanoparticle delivery device for the natural immunostimulatory milieu present in bacterial OMVs already, the normal immunosuppressive function of the probiotic bacteria is replaced with a powerful adjuvanting effect. Figure S2, Further comparative analysis of EcN and EcJ OMV vaccine formulations. (A) Dynamic light scattering hydrodynamic z-average particle sizes of EcJ and EcN OMVs (formulations assessed in PBS). (B) OMV zeta potentials assessed in PBS. (C) Dynamic light scattering hydrodynamic z-average particle sizes of EcN and EcN-lpxM OMVs (formulations assessed in PBS). (D) GFP fluorescence-standardized ClyA-GFP(+) vaccine doses of EcN and EcN-lpxM OMVs assayed for total protein content via BCA assay. #No significant difference (P>0.05). All values are given as mean +/− SD. Figure S3, EcN lpxm mutation does not result in detrimental loss of robust humoral immunity stimulation in a mouse model. Terminal titers of antigen-specific IgG from BALB/c mice vaccinated (primed) and boosted once with antigen-normalized doses (n = 5, each group). Experimental groups indicated are as follows: mice injected with recombinant GFP in PBS alone, GFP; with EcN OMVs from ECN containing the lpxM mutation, displaying ClyA-GFP, EcN-LpxM OMV-GFP; with EcN OMVs from non-lpxM mutant EcN, displaying ClyA-GFP, EcN OMV-GFP; with a mixture of recombinant ClyA-GFP and alum, Alum+ClyA-GFP. **P<0.001. (DOCX) [file pone.0112802.s001.docx]

**Supplemental Figures**

**
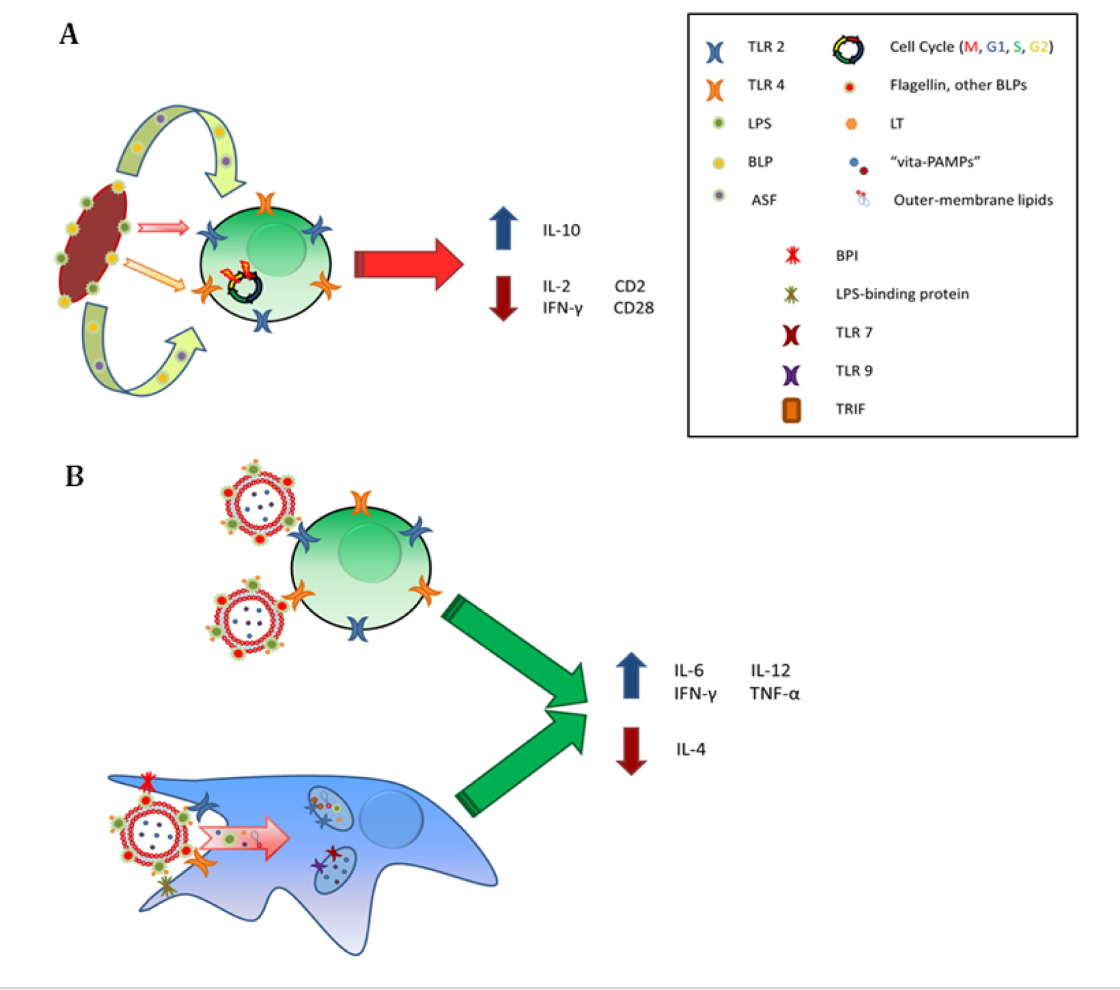
**

**Supplemental Figure S1| Isolation of EcN immunostimulatory factors from immunosuppressive ones via OMV formation leads to an induction of a strong immune response.**

(A) Natural immunosuppressive function of Nissle 1917 *E. coli* (EcN), focusing on direct effector action on αβ T-lymphocytes. Membranous, strain-specifically glycosylated bacterial lipopolysaccharide (LPS) and bacterial lipoproteins (BLPs), as well as secreted BLPs and other assorted secreted factors (ASFs), target the T-cells and transduce modulatory signals through TLRs TLR-2 and TLR-4 (S1–S3). These signals cause a variety of immunosuppressive effects that directly target the T-cells themselves (such as a decreased propensity to enter G2 or M phases of the cell cycle) (S2) as well as attenuate immunostimulatory and inflammatory activities through decreased secretion of cytokines (IL-2 and IFN-ɣ) and costimulatory molecules (CD2 and CD28) (S2). Direct immunosuppressive function is also upregulated by increased IL-10 secretion (S2).

(B) By isolating the targeting and immunostimulatory potential of the EcN membrane from EcN’s secretory immunosuppressive capabilities (S2, S3), and turning it into a bionanoparticle delivery device for the natural immunostimulatory milieu present in bacterial OMVs already (S1), the normal immunosuppressive function of the probiotic bacteria is replaced with a powerful adjuvanting effect. Specifically, these EcN OMVs take advantage of TLR-2 and TLR-4 targeting directly to T-cells (top) described previously as well as supplemented targeting to antigen-presenting cells (APCs) such as macrophages (bottom). In addition to TLR-mediated phagocytosis, macrophage cell membranes additionally contain bactericidal/permeability-increasing protein (BPI) and LPS-binding protein to further enhance phagocytosis-inducing avidity of membranous flagellin, BPIs, and LPS that bacterial OMVs are naturally enriched with (S1–S4). These interactions not only directly stimulate these immune cells through the binding event, but also facilitate delivery of a variety of immunostimulatory factors sensed intracellularly, such as TLR-4- and TRIF-activating LPS, heat-labile enterotoxin (LT), and outer-membrane lipids (S4, S5), and TLR-7- and TLR-9-activating “vita-PAMPs” (molecules that trick the immune cell into thinking a live pathogen is inside it) such as dsDNA, cytosolic mRNA, and bacterial proteases (S5, S6) . Taken together, the extracellular and intracellular immunostimulatory factors induce an enhanced inflammatory and generally immunoactivated state, which is spread to other immune cells through elevated IL-6, IL-12, IFN-ɣ, and TNF-α secretion, coupled with decreased IL-4 secretion (S1, S7, S8).


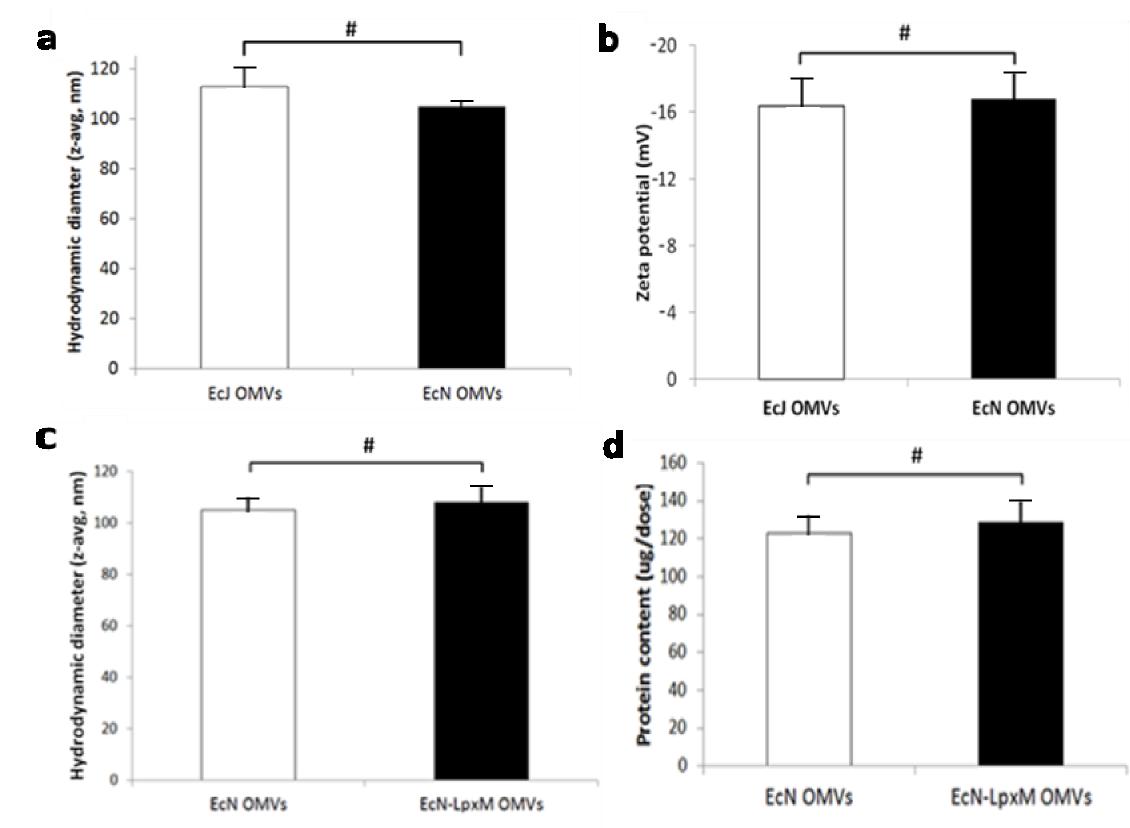


**Supplemental Figure S2 | Further comparative analysis of EcN and EcJ OMV vaccine formulations**. **a,** Dynamic light scattering hydrodynamic z-average particle sizes of EcJ and EcN OMVs (formulations assessed in PBS). **b,** OMV zeta potentials assessed in PBS. **c,** Dynamic light scattering hydrodynamic z-average particle sizes of EcN and EcN-*lpxM* OMVs (formulations assessed in PBS). **d,** GFP fluorescence-standardized ClyA-GFP(+) vaccine doses of EcN and EcN-*lpxM* OMVs assayed for total protein content via BCA assay. ^#^No significant difference (P>0.05). All values are given as mean +/- SD.

**
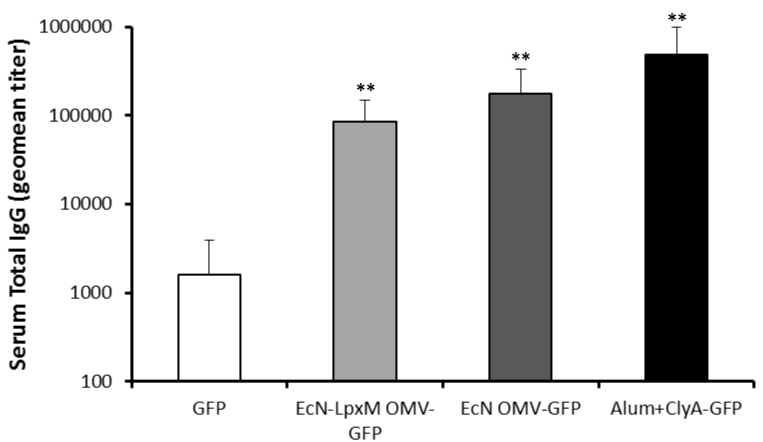
**

**Supplemental Figure S3 | EcN *lpxm* mutation does not result in detrimental loss of robust humoral immunity stimulation in a mouse model.** Terminal titers of antigen-specific IgG from BALB/c mice vaccinated (primed) and boosted once with antigen-normalized doses (n=5, each group). Experimental groups indicated are as follows: mice injected with recombinant GFP in PBS alone, GFP; with EcN OMVs from ECN containing the *lpxM* mutation, displaying ClyA-GFP, EcN-LpxM OMV-GFP; with EcN OMVs from non-lpxM mutant EcN, displaying ClyA-GFP, EcN OMV-GFP; with a mixture of recombinant ClyA-GFP and alum, Alum+ClyA-GFP. **P<0.001.

**Supporting Information Legends**

**Supplemental Figure S1| Isolation of EcN immunostimulatory factors from immunosuppressive ones via OMV formation leads to an induction of a strong immune response.**

(A) Natural immunosuppressive function of Nissle 1917 *E. coli* (EcN), focusing on direct effector action on αβ T-lymphocytes. Membranous, strain-specifically glycosylated bacterial lipopolysaccharide (LPS) and bacterial lipoproteins (BLPs), as well as secreted BLPs and other assorted secreted factors (ASFs), target the T-cells and transduce modulatory signals through TLRs TLR-2 and TLR-4 (S1–S3). These signals cause a variety of immunosuppressive effects that directly target the T-cells themselves (such as a decreased propensity to enter G2 or M phases of the cell cycle) (S2) as well as attenuate immunostimulatory and inflammatory activities through decreased secretion of cytokines (IL-2 and IFN-ɣ) and costimulatory molecules (CD2 and CD28) (S2). Direct immunosuppressive function is also upregulated by increased IL-10 secretion (S2).

(B) By isolating the targeting and immunostimulatory potential of the EcN membrane from EcN’s secretory immunosuppressive capabilities (S2, S3), and turning it into a bionanoparticle delivery device for the natural immunostimulatory milieu present in bacterial OMVs already (S1), the normal immunosuppressive function of the probiotic bacteria is replaced with a powerful adjuvanting effect. Specifically, these EcN OMVs take advantage of TLR-2 and TLR-4 targeting directly to T-cells (top) described previously as well as supplemented targeting to antigen-presenting cells (APCs) such as macrophages (bottom). In addition to TLR-mediated phagocytosis, macrophage cell membranes additionally contain bactericidal/permeability-increasing protein (BPI) and LPS-binding protein to further enhance phagocytosis-inducing avidity of membranous flagellin, BPIs, and LPS that bacterial OMVs are naturally enriched with (S1–S4). These interactions not only directly stimulate these immune cells through the binding event, but also facilitate delivery of a variety of immunostimulatory factors sensed intracellularly, such as TLR-4- and TRIF-activating LPS, heat-labile enterotoxin (LT), and outer-membrane lipids (S4, S5), and TLR-7- and TLR-9-activating “vita-PAMPs” (molecules that trick the immune cell into thinking a live pathogen is inside it) such as dsDNA, cytosolic mRNA, and bacterial proteases (S5, S6) . Taken together, the extracellular and intracellular immunostimulatory factors induce an enhanced inflammatory and generally immunoactivated state, which is spread to other immune cells through elevated IL-6, IL-12, IFN-ɣ, and TNF-α secretion, coupled with decreased IL-4 secretion (S1, S7, S8).

**Supplemental Figure S2 | Further comparative analysis of EcN and EcJ OMV vaccine formulations**. **a,** Dynamic light scattering hydrodynamic z-average particle sizes of EcJ and EcN OMVs (formulations assessed in PBS). **b,** OMV zeta potentials assessed in PBS. **c,** Dynamic light scattering hydrodynamic z-average particle sizes of EcN and EcN-*lpxM* OMVs (formulations assessed in PBS). **d,** GFP fluorescence-standardized ClyA-GFP(+) vaccine doses of EcN and EcN-*lpxM* OMVs assayed for total protein content via BCA assay. ^#^No significant difference (P>0.05). All values are given as mean +/- SD.

**Supplemental Figure S3 | EcN *lpxm* mutation does not result in detrimental loss of robust humoral immunity stimulation in a mouse model.** Terminal titers of antigen-specific IgG from BALB/c mice vaccinated (primed) and boosted once with antigen-normalized doses (n=5, each group). Experimental groups indicated are as follows: mice injected with recombinant GFP in PBS alone, GFP; with EcN OMVs from ECN containing the *lpxM* mutation, displaying ClyA-GFP, EcN-LpxM OMV-GFP; with EcN OMVs from non-lpxM mutant EcN, displaying ClyA-GFP, EcN OMV-GFP; with a mixture of recombinant ClyA-GFP and alum, Alum+ClyA-GFP. **P<0.001.

**Supplemental References**

S1. Granoff, D. M. 2010. Review of meningococcal group B vaccines. *Clin. Infect. Dis.* 50 Suppl 2: S54–65.

S2. Trebichavsky, I., I. Splichal, V. Rada, and A. Splichalova. 2010. Modulation of natural immunity in the gut by Escherichia coli strain Nissle 1917. *Nutr. Rev.* 68: 459–64.

S3. Grabig, A., D. Paclik, C. Guzy, A. Dankof, D. C. Baumgart, J. Erckenbrecht, B. Raupach, U. Sonnenborn, J. Eckert, R. R. Schumann, B. Wiedenmann, a U. Dignass, and A. Sturm. 2006. Escherichia coli strain Nissle 1917 ameliorates experimental colitis via toll-like receptor 2- and toll-like receptor 4-dependent pathways. *Infect. Immun.* 74: 4075–82.

S4. Zídek, Z., E. Kmonícková, P. Kostecká, and H. Tlaskalová-Hogenová. 2010. Decisive role of lipopolysaccharide in activating nitric oxide and cytokine production by the probiotic Escherichia coli strain Nissle 1917. *Folia Microbiol. (Praha)* 55: 181–9.

S5. Blasius, A. L., and B. Beutler. 2010. Intracellular toll-like receptors. *Immunity* 32: 305–15.

S6. Sander, L. E., M. J. Davis, M. V Boekschoten, D. Amsen, C. C. Dascher, B. Ryffel, J. a Swanson, M. Müller, and J. M. Blander. 2011. Detection of prokaryotic mRNA signifies microbial viability and promotes immunity. *Nature* 0–7.

S7. Guzy, C., D. Paclik, A. Schirbel, U. Sonnenborn, B. Wiedenmann, and A. Sturm. 2008. The probiotic Escherichia coli strain Nissle 1917 induces gammadelta T cell apoptosis via caspase- and FasL-dependent pathways. *Int. Immunol.* 20: 829–40.

S8. Bickert, T., C. M. Trujillo-Vargas, M. Duechs, G. Wohlleben, T. Polte, G. Hansen, T. a Oelschlaeger, and K. J. Erb. 2009. Probiotic Escherichia coli Nissle 1917 suppresses allergen-induced Th2 responses in the airways. *Int. Arch. Allergy Immunol.* 149: 219–30.
